# Deep Learning-Based Precision Analysis for Acrosome Reaction by Modification of Plasma Membrane in Boar Sperm

**DOI:** 10.3390/ani13162622

**Published:** 2023-08-14

**Authors:** Mira Park, Heemoon Yoon, Byeong Ho Kang, Hayoung Lee, Jisoon An, Taehyun Lee, Hee-Tae Cheong, Sang-Hee Lee

**Affiliations:** 1 School of Information and Communication Technology, University of Tasmania, Hobart, TAS 7005, Australia; 2 College of Animal Life Sciences, Kangwon National University, Chuncheon 24341, Republic of Korea; 3 College of Veterinary Sciences, Kangwon National University, Chuncheon 24341, Republic of Korea

**Keywords:** acrosome reaction, automatic detection, deep learning, pig, sperm, plasma membrane

## Abstract

**Simple Summary:**

The acrosome reaction (AR) is one of the important factors in assessing sperm infertility. However, the accuracy of these assessments may be influenced by the subjective judgments of experts. Addressing the issue of subjectivity in the assessment of the AR, we developed the Acrosome Reaction Classification System (ARCS). This system enables automatic calculation of the AR ratio using deep learning, which not only detects AR sperm by identifying micro-changes in the plasma membrane (PM), but also offers improved speed and performance compared to experts. Moreover, we established the need for independent ARCS with appropriate magnifications to detect AR sperm across various magnifications. The ARCS also offers consistent analysis for AR sperm detection and reduces misrecognition due to human error. In conclusion, our proposed methodology has the potential to contribute to the development of deep learning-based diagnostic models for sperm characteristics in pigs and other species, while the ARCS can be utilized in artificial intelligence-based infertility diagnoses within reproductive medicine.

**Abstract:**

The analysis of AR is widely used to detect loss of acrosome in sperm, but the subjective decisions of experts affect the accuracy of the examination. Therefore, we develop an ARCS for objectivity and consistency of analysis using convolutional neural networks (CNNs) trained with various magnification images. Our models were trained on 215 microscopic images at 400× and 438 images at 1000× magnification using the ResNet 50 and Inception–ResNet v2 architectures. These models distinctly recognized micro-changes in the PM of AR sperms. Moreover, the Inception–ResNet v2-based ARCS achieved a mean average precision of over 97%. Our system’s calculation of the AR ratio on the test dataset produced results similar to the work of the three experts and could do so more quickly. Our model streamlines sperm detection and AR status determination using a CNN-based approach, replacing laborious tasks and expert assessments. The ARCS offers consistent AR sperm detection, reduced human error, and decreased working time. In conclusion, our study suggests the feasibility and benefits of using a sperm diagnosis artificial intelligence assistance system in routine practice scenarios.

## 1. Introduction

Sperm infertility diagnosis is accompanied by laborious manual work that requires accurate assessment of thousands from billions of sperm [1]. The accuracy of sperm assessment improves with high resolving power and magnification, but these conditions increase the time to evaluate a representative sample of spermatozoa [1]. For this reason, experts select the appropriate magnification to detect infertile sperm in consideration of the working speed and microscopic resolving power. However, even for experts, it is very difficult to distinguish infertile sperm on the basis of micro-modifications of organelles at these magnifications. Unfortunately, these detection methods rely on subjective decisions of experts, which lead to a reduction in accuracy for acrosome-reacted sperm diagnosis.

The sperm head is covered with a plasma membrane (PM) and contains an acrosome and a nucleus [2]. In particular, the PM not only plays a role to protect organelles from the external environment but also protects the acrosome from external factors such as microorganisms and reactive oxygen species [3]. The acrosome reaction (AR) is the controlled release of the acrosome according to the status of the PM, and it occurs when the sperm encounters oocytes. Checking whether the AR process occurs properly is one of the key examinations used to evaluate sperm characteristics in the field of reproductive medicine. If the sperm undergoes the AR prematurely during ejaculation, it indicates damage to the PM in male reproductive organs, which is considered a reproductive problem [1]. Therefore, the morphological features of the PM and the acrosome are used to assess male infertility according to the guidelines set by the World Health Organization (WHO) [1].

From a reproductive biology perspective, when sperm is exposed to high levels of bicarbonate and reactive oxygen species, the PM becomes loose and fragmented [3]. After the acrosome becomes detached from the sperm head, this indicates that the AR of the sperm has occurred [1]. Generally, when the AR is released from the sperm head, it can be easily detected using an optical microscope with simple staining. However, when the acrosome is still remaining on the sperm head and the PM is damaged, identification is more challenging. Due to this difficulty, the examination of membrane-impermeable fluorescence dyes (MFDs) has been widely employed to detect the AR. [4]. MFD-based methods demonstrate high performance, particularly in detecting initial changes in the PM and acrosomal outer membrane. However, these methods require specialized and expensive equipment, making them primarily suitable for molecular biology research on sperm [5]. In contrast, xanthene–thiazine (Diff-Quick) and Coomassie Brilliant Blue (CBB) staining methods have been widely used in laboratories and hospitals for acrosome examination. These staining methods are simple and provide fast results in detecting the acrosome status compared to MFP methods. With low-magnification microscopy, these staining methods can identify the acrosome status within a few minutes. However, they may not effectively detect relatively small PM modification, making them unsuitable for detecting the initial AR. It is important to note that, although the detection of an accurate AR is one of the crucial methods for assessing male infertility in precision medicine, it still requires considerable labor and expensive equipment. Therefore, there is a need to develop a fast yet accurate technique for detecting the accurate AR using a standard optical microscope and simple staining methods.

Visual assessment of sperm AR is typically performed manually by experts, heavily relying on their subjective judgment. Additionally, the manual process has a low throughput as it involves inspecting only tens of cells from a specimen containing tens of millions. To address these issues, research on automatic sperm detection and classification in computer vision has been conducted for decades. Various studies have focused on threshold-based segmentation, edge detection [6,7], region-based segmentation [8,9], and snake algorithms [10,11]. However, these algorithms still have limitations in detecting features related to the accurate AR, such as the loss, expansion, and breaking of the sperm’s PM, especially across diverse magnification. Therefore, there is a need to develop a technique that can objectively classify the accurate AR on the basis of a visual assessment under different magnifications.

Object detection in computer vision has gained significant attention in recent years, mainly due to the remarkable progress in convolutional neural networks (CNNs) [12] and their region-based counterparts [13]. Among the notable advantages of CNN is its ability to generate effective feature representations of input data and accurately classify target classes [14]. Training a CNN model typically involves a considerable amount of computation due to the large number of trainable parameters. However, with the advancement of graphic processor units (GPUs) for training CNN models, the utilization of CNN models has become more efficient [12]. Furthermore, alongside the progress in CNN, object detectors have been developed to complement the advancements [13,15,16,17,18,19]. One widely used object detector is faster region-based CNN (Faster R-CNN), which employs a two-stage approach. [20]. In the first stage, Faster R-CNN uses a region proposal network (RPN) to generate regions of interests (ROIs); in the second stage, it performs object classification and bounding-box regression on the proposed regions [13]. Faster R-CNN can be implemented using various CNN architectures, and it is known that the depth of the CNNs closely influences both accuracy and speed [21]. They have demonstrated successful performance in various object detection tasks, including biological vision tasks such as leukocyte detection [22], mitochondrial localization [23], and tumor and cancer detection [24]. However, there are no studies on using Faster R-CNN for detecting AR in sperm. 

From a deep learning perspective, the accuracy and precision of a CNN architecture are typically improved by increasing the number of the hidden layers [21]. However, this improvement comes at the cost of reduced inference speed and increased requirement of computational power [21]. Therefore, selecting a suitable CNN architecture on the basis of the characteristics of the image is a strategy to enhance the performance of the trained model [25]. The Inceptions architecture (Inceptions) [26] and residual neural networks (ResNets) [27] are widely used as backbones for Faster R-CNN. These architectures incorporate techniques such as dimension regulation and skipping certain layers to improve training efficiency in deep learning. Additionally, the combination of inception architecture with residual connections, known as Inception–ResNet, has been proposed to reduce errors in computer vision tasks [28]. Although the detection speed of Inception–ResNet is slower compared to ResNet networks, it demonstrates strong performance in image detection. As most microscopic cellular images are in the form of still images, Inception–ResNet with Faster R-CNN shows great potential for detecting cellular characteristics in these images [29].

The number of studies utilizing deep learning for sperm classification and object detection in microscopic images has been steadily increasing. In particular, deep learning-based studies focusing on DNA status classification [30] and head abnormality detection [31] have demonstrated superior capabilities compared to a traditional computer vision method. These deep learning models [30,31] exhibit high performance in classifying morphological characteristics in microscopic images containing a single sperm. However, there is currently a lack of research on object detection based on deep learning in microscopic images containing dozens of sperm. Recently, attempts have been made to detect and locate sperms using CNN, but these studies were not able to accurately classify the morphological characteristics of sperm in microscopic images [32,33]. Therefore, there is a need for a system that can accurately detect the precise morphology of sperm in microscopic images containing dozens of sperm, which would assist in diagnosing male infertility through visual assessment. In this study, we propose a model that utilizes a CNN-based object detection and classification approach to replace the tedious tasks involved in both sperm detection in the specimen and visual assessment of the AR of sperm. 

## 2. Materials and Methods

### 2.1. Experimental Design

**Figure 1:**
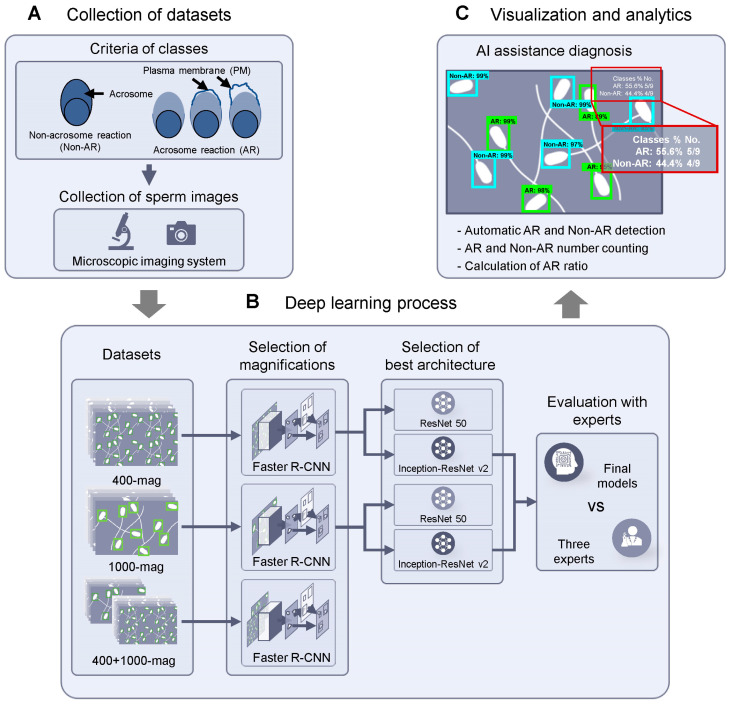
Scheme of Acrosome Reaction Classification System (ARCS): (**A**) Images containing AR/non-AR sperms are collected using a microscopic imaging system, and labeling data are annotated according to AR criteria. (**B**) Models are trained on the 400× (400-mag), 1000× (1000-mag) magnifications, and mixed 400-mag and 1000-mag (400 + 1000-mg) datasets, and datasets are trained using ResNet 50 and Inception–ResNet v2 architectures. (**B**) Selected models are compared with three experts. (**C**) Information of detected objects are visualized on the test images.

We developed an Acrosome Reaction Classification System (ARCS) that comprised three main steps. The first step involved collecting datasets at two magnifications: 400× (400-mag) and 1000× (1000-mag). Images containing both AR and non-AR boar sperms were collected using a microscopic imaging system (Figure 1A). In the second step, a deep learning process was performed. The labeling datasets consisted of 215 images at 400-mag (2732 AR and 1741 non-AR), 438 images at 1000-mag (2385 AR and 996 non-AR), and a mix of 400- and 1000-mag (653 images at 400 + 1000 mag, 5117 AR and 2737 non-AR). These datasets were trained using Faster R-CNN with the ResNet 50 architecture. Subsequently, the selected 400-mag and 1000-mag datasets were further trained using Inception–ResNet v2 to determine the best architecture (Figure 1B). Finally, the trained models using Inception–ResNet v2 were evaluated by three experts in a comparative manner. The third step involved the application of a user interface. The number of sperms and the automatically calculated AR ratios by ARCS were visualized on the microscopic images (Figure 1C).

### 2.2. Sperm Preparation and Dataset Collection

**Figure 2:**
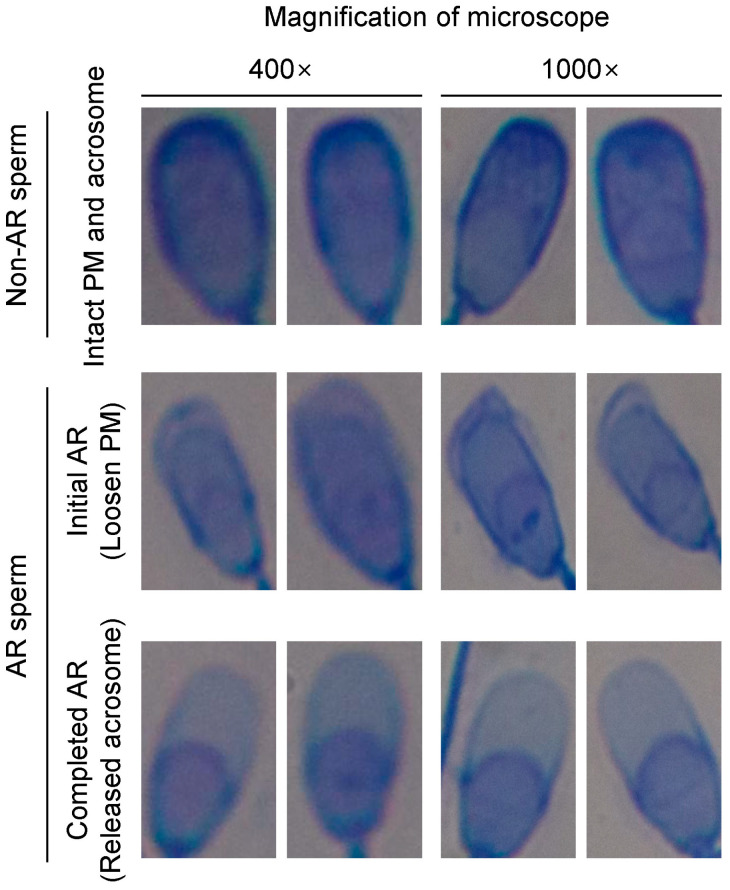
Criteria of acrosome reaction (AR) under 400× (400-mag) and 1000× (1000-mag) magnification. Non-AR sperm are shown with an acrosome that is full in the upper head (purple) and a plasma membrane (PM) that is intact. Initial AR sperms exhibit swelling of PM, while the acrosome remains intact. In the completed AR sperm, a significant loss of acrosome is observed.

We conducted all experiments in compliance with the scientific and ethical regulations, as followed by the Animal Experiment Ethics Committee at Kangwon National University, Republic of Korea (KIACUC-09-0139). Semen samples were collected from the pigs (n = 10, ages: 28.5 ± 6.2 months) using the glove-hand method. The samples were then diluted with semen extender (glucose 30.0 g/L, EDTA 2.25 g/L, sodium citrate 2.50 g/L, sodium bicarbonate 1.00 g/L, tris 5.00 g/L, citric acid 2.50 g/L, cysteine 0.05 g/L, gentamicin sulfate 0.30 g/L) to achieve a concentration of 1.5 × 10^7^ sperm/mL. To prepare the samples for detecting various AR sperm patterns, the diluted semen samples were centrifuged at 410 g for 5 min. The supernatant was removed, and the pellets were resuspended in 0.1 M phosphate buffer solution (PBS) to obtain a concentration of 1.5 × 10^7^ sperm/mL. Following a previous study [32], the samples were treated with 30 mM methyl-beta-cyclodextrin (MBCD, Sigma, St. Louis, MO, USA) for 30 min at room temperature. Subsequently, the semen samples were washed three times with PBS and resuspended in 0.1 M PBS to achieve a concentration of 1.5 × 10^7^ sperm/mL. For sample preparation, the samples were smeared onto slide glasses (Sigma) and dried. They were then washed three times with distilled water. After air-drying, the samples were stained with a 0.25% Coomassie Brilliant Blue (CBB, Sigma) solution for 5 min and dried at room temperature. Following that, the samples were washed three times with distilled water and dried again. Digital images of the samples were captured using a digital camera (EOS 750D, Canon, Tokyo, Japan) mounted on an optical microscope (BX50, Olympus, Tokyo, Japan). In terms of sperm classification, we considered intact PM and acrosomes as non-AR sperm, while swollen PM and released acrosomes were classified as AR sperm (Figure 2).

### 2.3. Data Preparation

We obtained a total of 653 images from the sperm samples of the 10 pigs, each image having dimensions of 1200 × 800 pixels (Table 1). Out of these, 215 images were magnified 400 times their actual size, while the remaining 438 images were magnified 1000 times their actual size. These magnifications, referred to as the 400-mag and 1000-mag images, respectively, are commonly used in the analysis of sperm microscopic images. All the images were in 24-bit RGB color format and saved as JPG files. To establish the ground truth for the images, three experienced embryologists with more than five years of research experience manually drew rectangular bounding boxes around the AR and non-AR sperms using an annotation tool [33]. The embryologists were instructed to draw tight bounding boxes around the head portion of the sperm, excluding any truncated portions that exceeded half the size of the sperm head if they extended beyond the image edges. Initially, the images were labeled independently by the three embryologists, and samples with a consensus agreement among at least two of the three experts were retained. The 400-mag images had a total of 2732 AR and 1741 non-AR ground-truth bounding boxes, while the 1000-mag images had 2385 AR and 996 non-AR bounding boxes (Table 1).

### 2.4. Model Training Using Convolutional Neural Networks (CNNs)

For the 400-mag images, a random split was performed, with 215 images allocated to the training dataset (172 images) and the test dataset (43 images). Similarly, for the 1000-mag images, a random split was conducted, resulting in 438 images assigned to the training dataset (351 images) and the test dataset (87 images) (Table 2). To achieve effective sperm object detection using the ARCS, it is essential to determine the hyperparameters associated with the trained models and the CNN architecture for object detection. In this study, a 5-fold cross-validation approach was employed. The entire training/validation process was repeated five times, with each iteration involving the rotation of a different 20% segment of the training dataset to form the validation set (Table 2). Data augmentation techniques were applied to enhance the training process. Specifically, a random vertical or horizontal flip was independently applied to 50% of the images in the training dataset during the training of the object detection model. Additionally, random rotations of 90°, as well as adjustments in brightness (with delta = 0.2), contrast (with 0.7 < delta < 1.1), and saturation (with 0.8 < delta < 1.25), were applied randomly to 50% of the images in the training datasets.

The procedure was implemented using TensorFlow 1.14.0 in Python 3.7.4. Two different CNN architectures, namely, ResNet 50 [27] and Inception–ResNet v2 [28], were considered for Faster R-CNN [13] to compare their performance. In order to address the class number imbalance, we utilized the linear inverse class frequency to regulate the weighted cross-entropy losses, as suggested in previous studies [34,35]. For the implementation of the sperm acrosome object detection, we employed the TensorFlow object detection package [36] and its extension. The Faster R-CNN model was pretrained on the COCO dataset [37] and then fine-tuned using the training dataset to detect AR/Non-AR. The training process was conducted on a machine equipped with a GPU (Geforce GTX 1080 Ti, NVIDIA, Santa Clara, CA, USA), and the operating system used was Ubuntu 16.04. 

The accuracy, precision, recall, F1 score, and mean average precision (mAP) at an intersection over union (IoU) of 0.5 were selected as measures to evaluate the performance of trained models. The IoU is defined as follows (1):

(1) $$\mathrm{IoU}=\frac{{\mathrm{Area}}_{\;\mathrm{Detected}\;\mathrm{box}}\cap \;{\mathrm{Area}\;}_{\mathrm{Ground}\;\mathrm{truth}}}{{\mathrm{Area}}_{\;\mathrm{Detected}\;\mathrm{box}}\cup {\mathrm{Area}\;}_{\mathrm{Ground}\;\mathrm{truth}}}$$

With an IoU threshold of 0.5, the accuracy (2), precision (3), and recall (4) were calculated, and the corresponding F1 score (5) could be defined as

(2) $$\mathrm{Accuracy}=\frac{\mathrm{TP}+\mathrm{TN}}{\mathrm{TP}+\mathrm{FP}+\mathrm{TN}+\mathrm{FN}}$$

(3) $$\mathrm{Precision}=\frac{\mathrm{TP}}{\mathrm{TP}+\mathrm{FP}}$$

(4) $$\mathrm{Recall}=\frac{\mathrm{TP}}{\mathrm{TP}+\mathrm{FN}}$$

(5) $$F1\;\mathrm{score}=\frac{2\times (\mathrm{Precision}\times \mathrm{Recall})}{\mathrm{Precision}+\mathrm{Recall}}$$

where true positive (TP) represents the number of objects detected with IoU > 0.5, false positive (FP) represents the number of detected boxes with IoU ≤ 0.5, false negative (FN) represents the number of objects that were not detected or detected with IoU ≤ 0.5, and true negative (TN) represents the number of objects that were misdetected with IoU ≤ 0.5. We also considered the FPS to check the efficiency of our approach.

The precision–recall curve is computed from a method’s ranked output, and recall is defined as the proportion of all positive examples ranked above a given rank [38]. The AP (6) summarizes the shape of the precision/recall curve, and is defined as follows [38]:

(6) $$AP=\frac{1}{11}\sum_{r\in \;\{0,0.1,\ldots ,1\}}P_{\mathrm{interp}}\left(r\right)$$

where $P_{interp}(r)$ (7) is the maximum precision for any recall values exceeding r [38]:

(7) $$P_{\mathrm{interp}}\left(r\right)=\underset{\tilde{r}:\tilde{r}\geq r}{\max}p(\tilde{r})$$

Lastly, the mAP (8) was calculated as an average of APs for all object classes:

(8) $$mAP=\frac{1}{N_{\mathrm{Class}}}\sum \mathrm{AP}$$

The performance results of the 5-fold cross-validation are presented as the mean ± standard deviation. The training and validation processes were treated as two separate procedures, and detection boxes were drawn using the trained models on the validation dataset with a score threshold of ≥0.8. The mean average precision (mAP) was evaluated on the validation dataset at every 500 iterations to fine-tune the hyperparameters. After several attempts, the following hyperparameters were determined: a maximum number of iterations of 30,000; an initial learning rate of 0.0003, which was then reduced to 0.00003 after 10,000 iterations. Subsequently, an experiment was conducted to determine the optimal CNN architecture for ARCS. The performances of the ResNet 50 and Inception–ResNet v2 architectures were measured and compared.

### 2.5. Comparison of Model Performance with Experts

After the completion of training and validation, the models were evaluated using the test dataset, which consisted of 43 (400-mag) and 87 (1000-mag) images. To assess the performance of the trained models, three expert embryologists (referred to as experts 1, 2, and 3) were involved in the evaluation. These experts did not see the image data prior to annotating the test dataset and possessed approximately 3 to 6 years of experience. The human annotation process followed the same rules as described in the previous section. To ensure accuracy, the experimenters thoroughly reviewed the annotations, correcting any potential mistakes before final submission. The annotations provided by the embryologists were then compared with the ground truth of the test dataset in order to calculate accuracy, precision, recall, and F1 scores. Furthermore, a comparison was made between the number of boxes assigned to the AR/non-AR classes and the AR sperm ratio, considering both the detected boxes by the trained models and the annotations provided by the three embryologists. This analysis was performed across all ranges of score thresholds and IoU values greater than 0.5.

### 2.6. Automatic Calculation of Acrosome Reaction Rate

The detected boxes of the AR/non-AR were drawn with the minimum score threshold r=0.8. The detected boxes were created, and the number of detected objects was counted by referring to the same index number of the array of detected classes. The total AR sperm ratio was calculated as follows (9):

(9) $$Total\;AR\;ratio=\frac{1}{N}\sum_{i=1}^{N}\frac{N_{AR}^{\left(i\right)}}{N_{AR}^{\left(i\right)}+N_{Non-AR}^{\left(i\right)}}\times 100$$

where *N* represents the number of images in the test dataset, and $N_{AR}^{\left(i\right)}$ and $N_{Non-AR}^{\left(i\right)}$ are the number of AR and non-AR boxes in the *i*-th image in the test dataset, respectively.

### 2.7. Statistical Analysis

Statistical analyses were performed using SAS v. 9.4 (SAS Institute, Cary, NC, USA). The ResNet-400-mag, ResNet-1000-mag, and ResNet-400 + 1000-mag datasets were evaluated using one-way analysis of variance (ANOVA). To compare the trained models based on ResNet 50 and Inception–ResNet v2, a t-test was employed. The results are presented graphically using a scatter dot plot generated with Graphpad Software Inc., San Diego, CA, USA, and the data are displayed as the mean ± standard error of the mean.

## 3. Results

### 3.1. Selection of Datasets According to Magnifications

**Figure 3:**
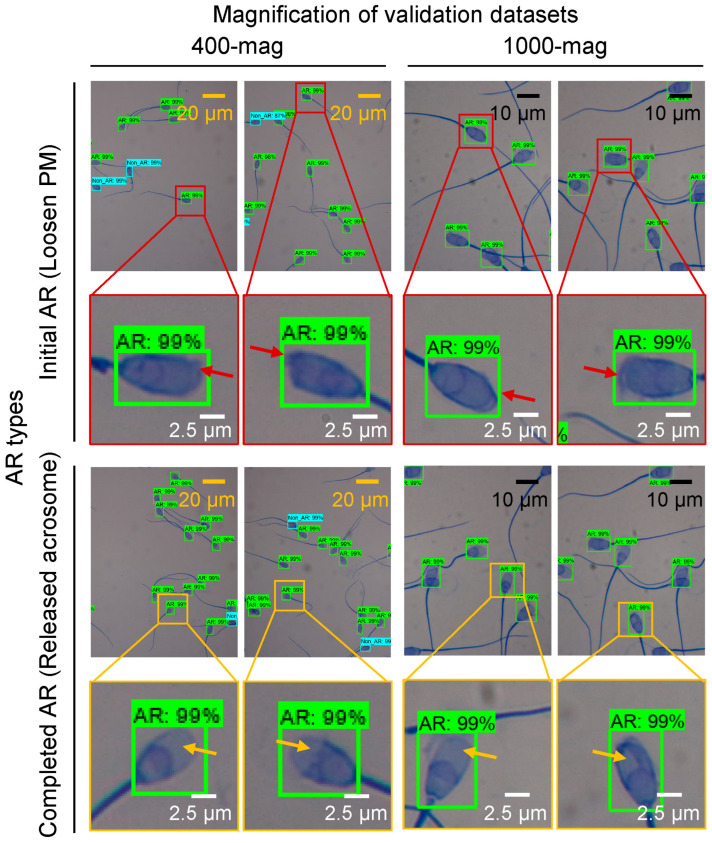
Detected acrosome reaction (AR) sperms under the 400× (400-mag) and 1000× (1000-mag) magnification by trained models. Initial AR sperm (red arrows) and completed AR sperm (yellow arrows).

ResNet-400-mag and ResNet-1000-mag classified the AR sperm when the validation datasets of the 400-mag and 1000-mag were evaluated (Figure 3). Specifically, the sperms exhibiting swollen PMs with retained acrosomes (initial AR, indicated by red arrows in Figure 3) were accurately detected in the validation datasets of both magnifications. Likewise, the sperms with released acrosomes (completed AR, indicated by yellow arrows in Figure 3) were successfully detected in the validation datasets of both magnifications.

**Figure 4:**
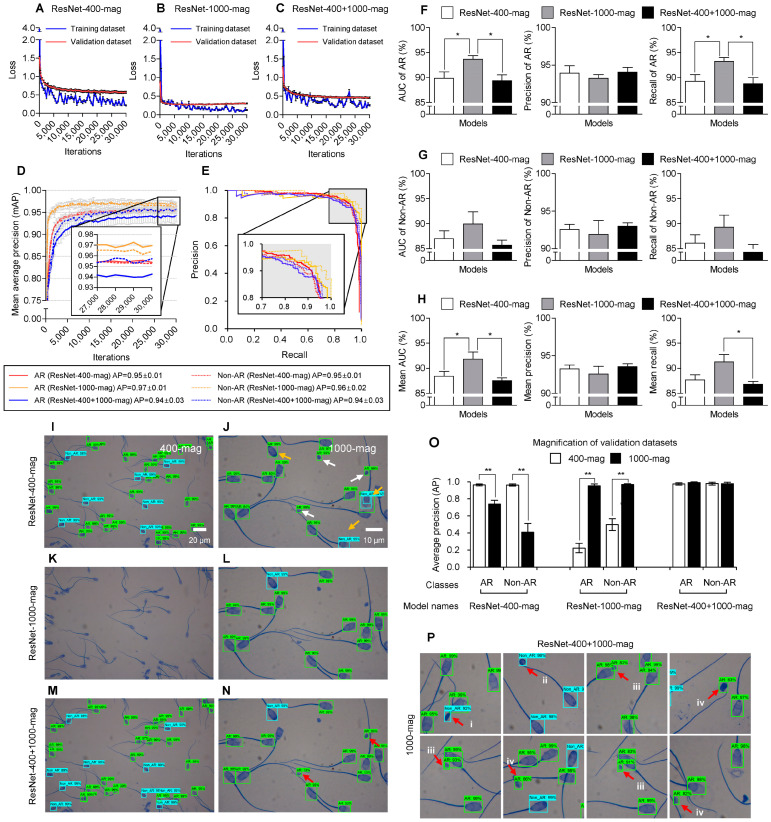
Comparison of performances in trained models according to 400× (400-mag), 1000× (1000-mag), and mixing of the 400× and 1000× (400+1000-mag) magnifications: (**A**–**C**) Learning curve of losses of the trained models on the 400-mag (ResNet-400-mag), 1000-mag (ResNet-1000-mag), and 400 + 1000-mag (ResNet-400 + 1000-mag) datasets during training. (**D**) Learning curve of mean average precision (mAP) in the ResNet-400-mag (red lines), ResNet-1000-mag (yellow lines), and ResNet-400 + 1000-mag (blue lines). (**E**) Precision–recall curve with the validation dataset by the trained models. (**F**–**H**) The accuracy (AUC), precision, and recall of the ResNet-400-mag, ResNet-1000-mag, and ResNet-400 + 1000-mag. Detected acrosome reaction (AR) and non-AR sperm by (**I**,**J**) ResNet-400-mag, (**K**,**L**) ResNet-1000-mag, and (**M**,**N**) ResNet-400 + 1000-mag in 400-mag and 1000-mag validation images. (**O**) Comparison of the APs among the trained models according to 400-mag (white bars) and 1000-mag (black bars) validation datasets. (**P**) Misrecognition of sperms (red arrows) by ResNet-400 + 1000-mag in 1000-mag: folded neck (i), coiled tail (ii), round type debris (iii), and broken heads (iv). The values of graphs are represented as the mean ± standard deviation in 5-fold cross-validation data and evaluated at 0.5 intersection over union (IoU). All models were trained using the ResNet 50 architecture. * *p* < 0.05, ** *p* < 0.01.

The models were trained on the training datasets of the 400-mag, 1000-mag, and 400 + 1000-mag using the ResNet 50 architecture, referred to as ResNet-400-mag, ResNet-1000-mag, and ResNet-400 + 1000-mag, respectively. Losses for both the training and the validation datasets stabilized after 10,000 iterations for all tested models. Notably, the ResNet-1000-mag exhibited the most stable learning curve, with the initial drop in loss falling below 0.5 (Figure 4A–C). The subsequent fluctuations in loss were less dramatic in the training dataset, indicating that the ResNet-1000-mag model had the best-fit learning curve.

The APs of the ResNet-400-mag, ResNet-1000-mag, and ResNet-400 + 100-mag models on the validation datasets (Figure 4D) stabilized after 10,000 iterations. In particular, the APs of the ResNet-1000-mag model (Figure 4D, represented by yellow lines) were higher than those of the other models. Within the ResNet-1000-mag model, the AP for AR (Figure 4D, yellow line) was higher than the AP for non-AR (Figure 4D, represented by a yellow dotted line). The AP for AR in the ResNet-1000-mag model (Figure 4E, yellow line, 0.97 ± 0.01) was higher compared to the ResNet-400-mag model (Figure 4E, red line, 0.95 ± 0.01) and the ResNet-400 + 1000-mag model (Figure 4E, blue line, 0.94 ± 0.03). Similarly, the AP for non-AR in the ResNet-1000-mag model (Figure 4E, yellow dotted line, 0.96 ± 0.02) was higher than in the other models. The accuracy (AUC) of AR (93.7 ± 1.6%) and the recall (93.2 ± 1.8%) in the ResNet-1000-mag model were significantly higher (*p* < 0.05) compared to the other models (Figure 4F, Table 3). Additionally, the mean accuracy of AR (91.8 ± 3.2%) in the ResNet-1000-mag model was significantly higher (*p* < 0.05) than in the other models (Figure 4H, Table 3). The accuracy, precision, recall, F1, and AP values for the AR class were higher than those for the non-AR class in the ResNet-400-mag, ResNet-1000-mag, and ResNet-400 + 1000-mag models (Table 3).

The ResNet-400-mag model successfully detected the AR (Figure 4I–N, indicated by green boxes) and non-AR (Figure 4I–N, indicated by blue boxes) in the validation datasets of both 400-mag and 1000-mag. However, in the validation dataset of 1000-mag, the ResNet-400-mag model mistakenly identified some debris as AR (Figure 4J, indicated by white arrows) and misclassified several AR as non-AR (Figure 4J, indicated by yellow arrows). On the other hand, the ResNet-1000-mag model failed to detect any sperm in the validation dataset of the 400-mag (Figure 4K), but accurately recognized AR and non-AR in the validation dataset of the 1000-mag (Figure 4L). Interestingly, the ResNet-400 + 1000-mag model successfully detected both AR and non-AR sperms in the validation datasets of both 400-mag (Figure 4M) and 1000-mag (Figure 4N). The APs of AR and non-AR in the ResNet-400-mag model were significantly higher (*p* < 0.01) in the 400-mag validation dataset compared to the 1000-mag validation dataset (Figure 4O). Conversely, the APs of AR and non-AR in the ResNet-1000-mag model were significantly lower (*p* < 0.01) in the 400-mag validation dataset compared to the 1000-mag validation dataset (Figure 4O). In contrast, the ResNet-400 + 1000-mag model demonstrated APs of over 94% for both AR and non-AR in both the 400-mag and 1000-mag validation datasets (Figure 4O). Despite achieving high APs (AR: 0.98 ± 0.01 and non-AR: 0.97 ± 0.01) when evaluating the validation dataset of 1000-mag (Figure 4O, represented by black bars in the ResNet-400 + 1000-mag group), the ResNet-400 + 1000-mag model consistently misclassified folded neck (Figure 4P, i), coiled tail (Figure 4P, ii), round-type debris (Figure 4P, iii), and broken heads (Figure 4P, iv) as sperms (Figure 4N,P, indicated by red arrows).

### 3.2. Selection of the Best Architecture

**Figure 5:**
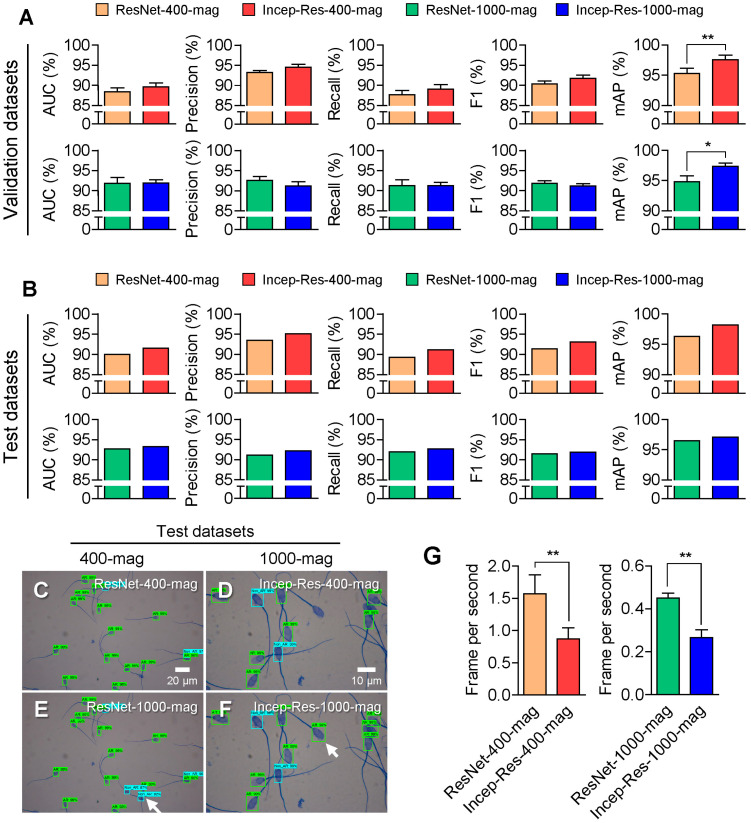
Comparison of ResNet 50 and Inception–ResNet v2 architectures on accuracy (AUC), precision, recall, F1, and mean average precision (mAP). (**A**,**B**) Metric results of the trained models on 400× (400-mag) and 1000× (1000-mag) magnification datasets based on ResNet 50 (ResNet-400-mag and ResNet-1000-mag) and Inception–ResNet v2 (ResNet-400 and Incep-Res-400-mag) using validation datasets (**A**) and test datasets (**B**). (**C**–**F**) Representative images of detected acrosome reaction (AR) and non-AR sperms by ResNet-400-mag (**C**), Incep-Res-400-mag (**D**), ResNet-1000-mag (**E**), and Incep-Res-1000-mag (**F**) in the 400-mag and 1000-mag datasets. White arrows indicate that detected boxes in the models based on Inception–ResNet v2 architecture. (**G**) Comparison of the frames per second in the trained models based on ResNet 50 and Inception–ResNet v2 architectures during evaluation of 43 images of the 400-mag and 87 images of the 1000-mag test datasets. The values of graphs are represented as the mean ± standard deviation in the 5-fold cross validation data and evaluated at 0.5 intersection over union (IoU). * *p* < 0.05, ** *p* < 0.01.

In this section, we employed the Inception–ResNet v2 architecture to enhance the training performance of the selected training datasets of 400-mag and 1000-mag. The trained model on the 400-mag training dataset based on Inception–ResNet v2 (Incep-Res-400-mag) exhibited higher accuracy, precision, recall, F1, and mAP compared to ResNet-400-mag (Figure 5A, Table 4). In particular, the mAP of Incep-Res-400-mag was significantly (*p* < 0.01) higher than that of ResNet-400-mag (Figure 5A). Additionally, the AUC, recall, and mAP of the trained model on the 1000-mag training dataset based on Inception–ResNet v2 (Incep-Res-1000-mag) are higher than those of ResNet-1000-mag (Figure 5A, Table 4). Moreover, Incep-Res-400-mag and Incep-Res-1000-mag detected more sperms than ResNet-400-mag and ResNet-1000-mag in the validation datasets of 400-mag and 1000-mag (Figure 5C–F, indicated by white arrows).

Next, we report the median mAP values of the models in the 5-fold cross-validation process. The accuracy, precision, recall, F1, and mAP were higher in Incep-Res-400-mag and Incep-Res-1000-mag than in ResNet-400-mag and ResNet-1000-mag in the test datasets (Figure 5B, Table 5). However, the frames per second (FPS) were significantly (*p* < 0.01) reduced by 1.75 times in Incep-Res-400-mag compared to ResNet-400-mag and by 1.69 times in Incep-Res-1000-mag compared to ResNet-1000-mag (Figure 5G, Table 5). Despite the decrease in FPS in the models based on the Inception–ResNet v2 architecture, other metrics were improved by Inception–ResNet v2 compared to the ResNet 50 architecture. On the basis of these results, we selected Incep-Res-400-mag and Incep-Res-1000-mag for comparison with the experts.

### 3.3. Comparison of Model Performances with Expert

**Figure 6:**
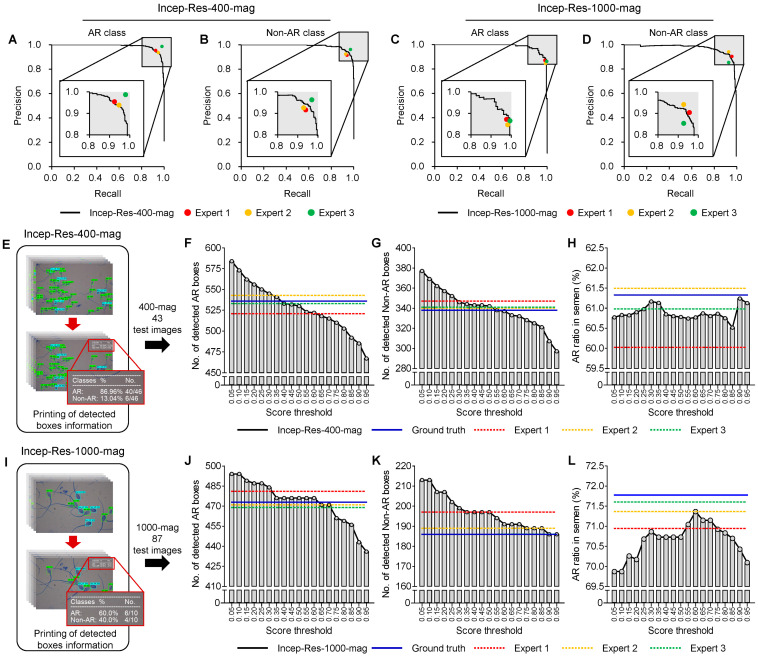
Comparison of precision–recall curve and calculated acrosome reaction (AR) ratios between trained models and experts in the test datasets: (**A**–**D**) The trained models on 400× (Incep-Res-400-mag) and 1000× (Incep-Res-1000-mag) magnification datasets based on Inception–ResNet v2 compared with three experts using precision–recall curve. (**E**,**I**) Presented boxes containing classes information, counted boxes, and calculated AR ratios on the test images. (**F**–**L**) The number of detected AR (**F**) and non-AR (**G**) in 400-mag by Incep-Res-400-mag, and the AR (**J**) and non-AR (**K**) in 1000-mag by Incep-Res-400-mag according to score thresholds. Comparison of the AR ratios among the trained models (black lines), ground truth (blue lines), expert 1 (red lines), expert 2 (yellow lines), and expert 3 (green lines) in the 400-mag (**H**) and 1000-mag (**L**) datasets.

The detection performances of Incep-Res-400-mag (Figure 6A, black line) were similar to those of expert 1 (Figure 6A, red point) and expert 2 (Figure 6A, yellow point), but lower than that of expert 3 (Figure 6A, green point) when the AR was detected. On the other hand, Incep-Res-400-mag (Figure 6B, black line) performed better than expert 1 (Figure 6B, red point) and expert 2 (Figure 6B, yellow point) in the non-AR class. The detection performance of the AR class in the Incep-Res-1000-mag (Figure 6C, black line) was higher than that of expert 1 (Figure 6C, red point) and expert 2 (Figure 6C, yellow point), and Incep-Res-1000-mag (Figure 6D, black line) classified the non-AR class better than expert 3 (Figure 6D, green point).

Lastly, we compared the automatically calculated AR ratios of Incep-Res-400-mag and Incep-Res-1000-mag with three experts. Additionally, to facilitate the analysis of AR through visual assessment, we printed information about the detected boxes on the images. As a result, the test images successfully display information about the classes, the number of detected sperm, and the AR ratio on the upper right side (Figure 6E,I, red boxes). The number of detected sperms by the Incep-Res-400-mag was similar to the ground truth (Figure 6F,G, blue lines) when the score thresholds were set to 0.45 (AR sperm, Figure 6F) and 0.55 (non-AR sperm, Figure 6G). Similarly, the number of detected boxes by the Incep-Res-1000-mag was comparable to the ground truth at score thresholds of 0.65 (AR sperm, Figure 6J) and 0.90 (non-AR sperm, Figure 6K). The range of calculated AR ratio using Incep-Res-400-mag was 60.77% to 61.13% (Figure 6H), while, for Incep-Res-1000-mag, it ranged from 69.87% to 70.10% (Figure 6L). Interestingly, the calculated AR ratios of both the Incep-Res-400-mag and the Incep-Res-1000-mag closely resemble the AR ratios of the ground truth in the 400-mag (61.33%) and the 1000-mag (71.78%) test datasets when the score thresholds were set to 0.90 (Figure 6H) and 0.60 (Figure 6L), respectively.

## 4. Discussion

The accuracy of cytological sperm diagnosis has advanced in conjunction with the development of microscopic resolving powers. However, despite these advancements, detecting micro-changes in organelles such as the PM, nucleus, and mitochondria still requires professional knowledge in the field of sperm analysis. Furthermore, to ensure the reliability of sperm analysis in experimental and clinical settings, hundreds or even thousands of specimens need to be evaluated. The requirement for specialized domain knowledge and the demanding nature of these tasks can lead to misidentification and subjective judgments by experts, resulting in inaccurate sperm analysis results. Our system consistently recognizes micro-changes in the PM of microscopic images on the basis of pixel information and correctly classifies them as AR sperm. Moreover, our system can effectively distinguish initial AR sperm even at 400-mag. This implies that our approach can objectively identify micro-modifications in the PMs of sperm, which are challenging for experts to diagnose accurately across numerous specimens at 400× magnification. Therefore, our system has the potential to replace manual labor in sperm diagnosis. Additionally, we intentionally did not utilize 100× and 200× magnification images for model training due to their low resolving power, making it difficult to discern micro-changes in the PM.

The seminal plasma comprises various inorganic substances, including white blood cells, microorganisms, and tissue and cell debris [39]. These substances hinder the visual assessment of sperm, which is why semen undergoes a washing process [5]. However, despite the washing process, some debris remains in the samples [40]. In practice, certain substances in the semen resemble sperm heads when observed under a microscope, posing a challenge to accurate sperm analysis. Therefore, a technique for detecting sperm heads is necessary for diagnosing AR sperm. In this study, we utilized datasets containing labeled images of sperm heads from 400-mag, 1000-mag, and a combination of both. However, when evaluating the model trained on the mixed 400-mag and 1000-mag datasets, we observed instances where the model mistakenly identified folded necks, coiled tails, broken heads, and rounded debris as sperm heads in the 1000-mag evaluation dataset. If we used labeled datasets that included the head, midpiece, and tail of sperm in our study, the model would have avoided misidentifying substances as sperm heads in the 1000-mag dataset. However, the training performance of the model would have been compromised compared to the model developed in this study. Additionally, semen contains diverse substances of various sizes and shapes, which can vary depending on male health and environmental factors [41]. Unfortunately, the diversity of these substances cannot be predicted prior to semen ejaculation because visual assessment of sperm cannot be performed. We recognize this as a current limitation of deep learning-based computer vision in the field of sperm research. Consequently, instead of employing image size augmentation and post-image processing to address this problem, we decided to exclude the ResNet-400 + 1000-mag model and developed two independent models for 400-mag and 1000-mag.

Sperm analysis based on visual assessment is broadly classified into two main methods: motility analysis and morphological analysis [4]. Motility analysis allows for the detection of overall movements but lacks the ability to detect detailed morphology due to constant microscopic focusing changes caused by sperm movement. On the other hand, morphological analysis is performed on fixed sperm and is useful for detecting detailed organelles. Therefore, experts must choose the appropriate method depending on the examination purpose. Similarly, selecting a suitable detector and CNN architecture is crucial for the successful development of sperm analysis based on deep learning. In this study, we used a two-stage detector to develop the ARCS because the sperm for AR detection is fixed on slide glass. Additionally, our focus was on accurate detection rather than inference speed. In practice, the Inception–ResNet v2-based ARCS outperformed ResNet 50 by reducing false negatives and false positives. This indicates that the inception blocks of Inception–ResNet v2 effectively trained the sperm head type. Furthermore, the Inception–ResNet v2-based ARCS demonstrated remarkable performance that surpassed embryologists in terms of calculation speed. These results imply that it enhanced analytical efficiency within a limited timeframe. As mentioned earlier, embryologists sometimes misidentify sperm due to heavy reliance on subjective choices. Interestingly, despite variations among the three embryologists in their detection abilities, the ARCS performed within their levels of performance. This suggests that the ARCS can overcome inter-variation in sperm analysis when handling thousands of specimens and calculating AR ratios from extensive datasets. Hence, it has the potential to replace the work of experts and reduce diagnosis time in detecting AR sperm using our approach.

Therefore, we developed the ARCS, which automatically calculates the AR ratio on the basis of deep learning. It not only detects AR sperm with micro-changes in the PM but also exhibits competitive speed and performance compared to experts. Additionally, we emphasize the need for independent ARCS tailored to appropriate magnifications for detecting AR sperm in various scenarios. Thus, our model can replace the tedious tasks of detecting sperm in numerous specimens and the subjective assessment of experts in determining the AR status of micro-changed PMs in sperm heads using CNN-based object detection and classification. Moreover, the ARCS contributes to consistent AR sperm detection and reducing human errors in misrecognition. 

## 5. Conclusions

In conclusion, the proposed methodology contributes to the development of a deep learning-based diagnostic model for detecting sperm characteristics in pigs and other species. The ARCS can be utilized in artificial intelligence-based infertility diagnosis in reproductive medicine.

## Figures and Tables

**Table 1:** Description of the experimental dataset from ten pigs of 400× (400-mag) and 1000× (1000-mag) magnification microscopic images.

Experimental Animal ID	No. of 400-mag Dataset			No. of 1000-mag Dataset		
	Images	AR Sperm	Non-AR Sperm	Images	AR Sperm	Non-AR Sperm
1	21	312	164	45	291	99
2	22	412	176	42	243	87
3	20	173	137	45	291	93
4	23	210	130	45	213	78
5	22	342	243	45	234	117
6	21	306	178	42	300	102
7	22	286	138	45	279	93
8	21	214	220	42	243	114
9	21	175	119	45	204	129
10	22	302	236	42	87	84
Total	215	2732	1741	438	2385	996

Acrosome reacted sperm, AR sperm; non-acrosome reacted sperm, non-AR sperm.

**Table 2:** Distribution of dataset for 5-fold cross validation in data of 400× (400-mag), 1000× (1000-mag), and mixing of 400× and 1000× (400 + 1000-mag) magnification images.

Dataset		Folds	Images	No. of Labeling Data	
				AR	Non-AR
400-mag	Training	Fold 1	34	421	273
		Fold 2	34	442	280
		Fold 3	34	418	274
		Fold 4	35	444	296
		Fold 5	35	469	279
	Test	-	43	538	339
1000-mag	Training	Fold 1	72	363	183
		Fold 2	69	387	153
		Fold 3	69	369	159
		Fold 4	69	399	141
		Fold 5	72	396	168
	Test	-	87	471	192
400 + 1000-mag	Training	Fold 1	106	784	456
		Fold 2	103	829	433
		Fold 3	103	787	433
		Fold 4	104	843	437
		Fold 5	107	865	447
	Test	-	130	1009	531

**Table 3:** Performance results on the accuracy, precision, recall, F1, and average precision (AP) with validation dataset in trained models according to microscopic magnifications.

Classes	Model Names	Accuracy	Precision	Recall	F1	AP
AR	ResNet-400-mag	0.899 ± 0.028	0.939 ± 0.022	0.893 ± 0.030	0.915 ± 0.020	0.954 ± 0.010
	ResNet-1000-mag	0.937 ± 0.016	0.932 ± 0.011	0.932 ± 0.018	0.932 ± 0.012	0.968 ± 0.014
	ResNet-400 + 1000-mag	0.894 ± 0.027	0.941 ± 0.013	0.888 ± 0.027	0.913 ± 0.013	0.943 ± 0.014
Non-AR	ResNet-400-mag	0.870 ± 0.035	0.926 ± 0.015	0.861 ± 0.037	0.892 ± 0.022	0.952 ± 0.012
	ResNet-1000-mag	0.899 ± 0.054	0.919 ± 0.042	0.893 ± 0.054	0.904 ± 0.019	0.964 ± 0.021
	ResNet-400 + 1000-mag	0.857 ± 0.023	0.930 ± 0.010	0.848 ± 0.023	0.887 ± 0.012	0.957 ± 0.004
Average	ResNet-400-mag	0.884 ± 0.021	0.932 ± 0.011	0.877 ± 0.023	0.903 ± 0.017	0.953 ± 0.008
	ResNet-1000-mag	0.918 ± 0.032	0.926 ± 0.023	0.912 ± 0.033	0.918 ± 0.015	0.966 ± 0.015
	ResNet-400 + 1000-mag	0.875 ± 0.013	0.935 ± 0.009	0.868 ± 0.013	0.900 ± 0.008	0.950 ± 0.021

AR, acrosome reacted sperm; non-AR, non-acrosome reacted sperm; ResNet-400mag, trained on 400× magnification image dataset; ResNet-1000mag, trained on 1000× magnification image dataset; ResNet-400 + 1000mag, trained on mixed 400× and 1000× magnification image dataset. All models consisted of Faster R-CNN with ResNet 50 architecture. Data are represented as the mean ± standard error mean.

**Table 4:** Comparison of ResNet 50 (ResNet) and Inception–ResNet v2 (Incep-Res) architectures according to the accuracy, precision, recall, F1, and mean average precision (mAP) of validation dataset in trained models on 400× (400-mag) and 1000× (1000-mag) magnification datasets.

Models	Accuracy	Precision	Recall	F1	mAP
ResNet-400-mag	0.884 ± 0.021	0.932 ± 0.011	0.877 ± 0.023	0.903 ± 0.017	0.953 ± 0.008
Incep-Res-400-mag	0.896 ± 0.022	0.945 ± 0.016	0.891 ± 0.024	0.917 ± 0.018	0.976 ± 0.007
ResNet-1000-mag	0.918 ± 0.032	0.926 ± 0.023	0.912 ± 0.033	0.918 ± 0.015	0.942 ± 0.026
Incep-Res-1000-mag	0.919 ± 0.018	0.912 ± 0.024	0.913 ± 0.018	0.912 ± 0.006	0.974 ± 0.005

Data are represented as the mean ± standard error mean. All experiments were conducted by 5-fold cross validation.

**Table 5:** Comparison of ResNet 50 (ResNet) and Inception–ResNet v2 (Incep-Res) architectures according to the accuracy, precision, recall, F1, mean average precision (mAP), and frames per second (FPS) of test dataset in trained models on 400× (400-mag) and 1000× (1000-mag) magnification datasets.

Models	Accuracy	Precision	Recall	F1	mAP	FPS
ResNet-400-mag	0.900	0.935	0.893	0.914	0.963	1.572 ± 0.132
Incep-Res-400-mag	0.916	0.951	0.912	0.931	0.982	0.872 ± 0.076
ResNet-1000-mag	0.927	0.911	0.920	0.915	0.965	0.450 ± 0.023
Incep-Res-1000-mag	0.933	0.922	0.927	0.919	0.971	0.266 ± 0.017

Data are represented as the mean ± standard error mean.

## Data Availability

All data generated or analyzed during this study are included in this published article.
